# Effects of low-dose intravenous heparin therapy in aneurysmal subarachnoid hemorrhage: a randomized controlled clinical trial protocol

**DOI:** 10.1186/s13063-023-07493-9

**Published:** 2023-07-08

**Authors:** Yifan Zhang, Jiangang Hu

**Affiliations:** 1 Department of Neurology, ShenZhen BaoAn People’s Hospital, ShenZhen, China; 2 Department of Neurosurgery, ShenZhen BaoAn People’s Hospital, ShenZhen, China

**Keywords:** Aneurysmal subarachnoid hemorrhage, Randomized controlled trial, RCT protocol

## Abstract

**Background:**

Heparin anticoagulation therapy is a widely used method to prevent cerebral vasospasm (CV) and venous thrombosis in patients with subarachnoid hemorrhage caused by ruptured cerebral aneurysms. Subcutaneous heparin injection is considered safe and effective, whereas continuous intravenous heparin infusion is still being debated due to the risk of bleeding. Although most retrospective studies have confirmed the safety and effectiveness of unfractionated heparin (UFH) after aneurysm embolization therapy and its ability to reduce CV, there is still no randomized clinical trial comparing UFH and subcutaneous low-molecular-weight heparin (LMWH) injection in this population. Therefore, this study aims to compare the clinical outcomes associated with these two treatment approaches.

**Methods:**

The study is an open-label, single-center, randomized controlled trial and aims to recruit 456 patients, with 228 patients in each group. The primary outcome was CV; the second outcomes measures are occurrence of bleeding events, ischemic events, heparin-induced thrombocytopenia, deep vein thrombosis, cerebral venous circulation time, brain edema score, and hydrocephalus incidence.

**Ethics and dissemination:**

This study protocol obtained ethical approval from the Ethics Committee of Baoan People’s Hospital, Shenzhen, Guangdong (approval number: BYL20220805). This work will be published in peer-reviewed international medical journals and presented at medical conferences.

**Trial registration:**

ClinicalTrials ID: NCT05696639. Registered on March 30, 2023.

**Supplementary Information:**

The online version contains supplementary material available at 10.1186/s13063-023-07493-9.

## Background

Aneurysmal subarachnoid hemorrhage (aSAH) is a highly fatal disease with a high incidence rate, accounting for about 80% of cases of SAH [1], and 46% of aSAH survivors suffer from long-term cognitive impairment [2]. Approximately one third of patients require lifelong care, which greatly affects their quality of life and places a tremendous burden on society [3].

Due to the complex pathophysiology of aSAH, it is often associated with ischemic and hemorrhagic complications. Although early treatment of aneurysms has become the primary means of preventing rebleeding, postoperative neurological complications are also common, including vasospasm, hydrocephalus, and rebleeding [4]. Cerebral vasospasm (CV) is the most severe and dangerous complication in the early stages of hemorrhage, typically occurring within 3–14 days following the onset of bleeding. The incidence of vascular spasm accounts for 40–70% of cases of aSAH and remains a significant factor contributing to morbidity and mortality [5]. It restricts blood flow and increases the risk of ischemia. Treatment options for CV include calcium channel blockers and endovascular therapy, as well as maintaining circulatory blood volume [6]. In addition to these treatments, the potential of heparin to reduce the incidence and mortality of aSAH-induced CV has gradually been explored, and it has also been shown to have anti-inflammatory and neuroprotective properties [7].

Unfractionated heparin (UFH) is a safe and commonly used anticoagulant, typically employed for the prevention of venous thrombosis and hypercoagulable states. It primarily functions by increasing the activity of antithrombin III to inhibit the activity of thrombin and factor Xa, thus preventing the formation of blood clots [8]. However, one of the side effects of heparin is bleeding, which necessitates coagulation function monitoring during its use.

In patients undergoing endovascular treatment for aSAH, continuous maintenance of arterial catheters with UFH during surgery contributes to the reduction of thrombus formation, while subcutaneous injections of low-molecular-weight heparin (LMWH) prevent formation of lower-extremity deep veinous thrombosis. However, both LMWH and UFH infusions have their pros and cons. Although neurocritical care guidelines recommend initiating subcutaneous heparin 24 h after aneurysm embolization surgery [9], the UFH injection has limitations due to its high molecular weight and low bioavailability. Intravenous UFH may, therefore, be considered more reliable.

Research indicates that low-dose intravenous UFH infusion yields favorable outcomes in reducing CV and ischemic complications. Multiple retrospective studies have demonstrated the safety and efficacy of low-dose continuous intravenous UFH infusion. However, there is controversy regarding the optimal dosage and duration of treatment. A retrospective study by J. Marc Simard revealed the safety of low-dose (8–9 U/kg/h) intravenous UFH administration in aSAH patients, without any cases of bleeding, heparin-induced thrombocytopenia (HIT), or deep vein thrombosis, and the incidence of clinical vasospasm significantly decreased compared to subcutaneous LMWH usage [10]. Another research by Markus Bruder in endovascular treatment for aSAH patients showed a lower probability of cerebral infarction when combining UFH with LMWH [11].

Regarding the timing of initiating of heparin, Annika’s study suggested that using heparin within 24 h after aneurysm repair might be safer and carry a lower risk of bleeding compared to initiating treatment 48 h later [12]. Randomized controlled trial results comparing subcutaneous injections of LMWH and intravenous infusion of UFH are currently lacking, although randomized controlled studies have shown the safe and effective reduction of CV and ischemia with subcutaneous injection of enoxaparin compared to placebo in patients with aSAH [13].

This study is a single-center exploratory randomized controlled trial comparing the impact of low-dose intravenous infusion of UFH and subcutaneous injection of LMWH on the incidence of CV in aSAH patients. The outcomes to be assessed also include the occurrence of bleeding events, ischemic events, HIT, deep vein thrombosis, cerebral venous circulation time, brain edema score, and hydrocephalus incidence.

### Trial status

The protocol version number for this experimental design is BYL20220216. The recruitment for the experiment is scheduled to commence in December 2023, with an estimated completion date for recruitment by December 2025.

## Methods

### Study design

**Fig. 1:**
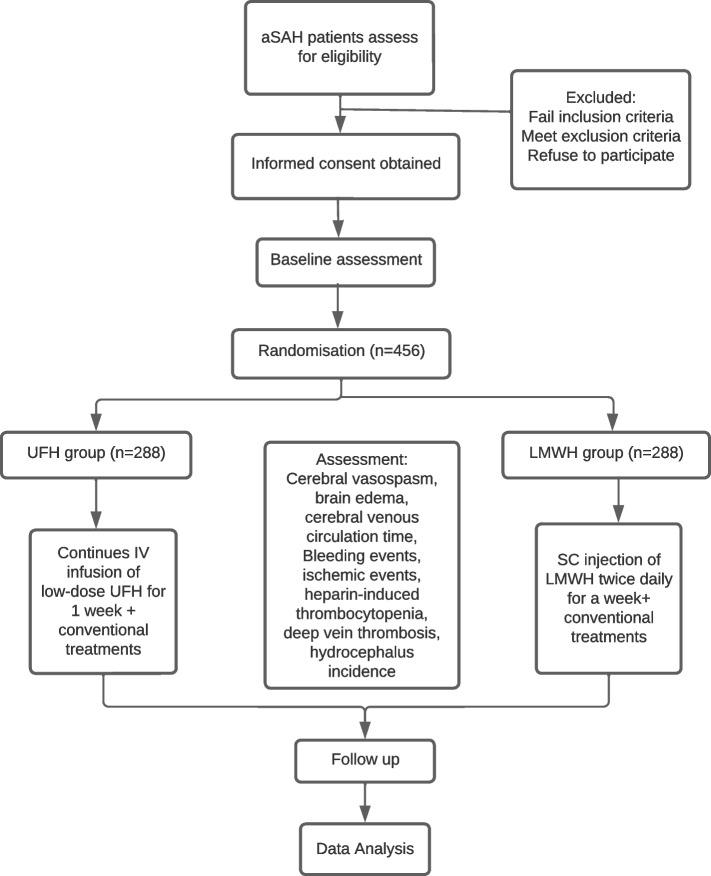
Flowchart of the trial

This is a prospective, single-center, double-blind, randomized controlled trial. The protocol has been approved by the Ethics Committee of Shenzhen Baoan People’s Hospital (BYL20220805) and registered on ClinicalTrials.gov (NCT05696639). The trial will be conducted at a tertiary teaching hospital in Shenzhen, Guangdong Province, China (Shenzhen Baoan People’s Hospital). This hospital covers an area of approximately 40,000 square meters and has more than 1000 beds, serving a population of approximately 580,000 million residents (data revised in 2020), and the study protocol follows the SPIRIT checklist. The schedule for participant enrollment, intervention, and assessment is based on the SPIRIT figure (Fig. 1).

### Participants

The recruitment program for participants is scheduled to commence in the year 2023 and is expected to conclude by December of 2025. Enrolled patients will be admitted to a neurosurgical monitoring ward and receive medical treatment according to the Guidelines for the Management of Aneurysmal Subarachnoid Hemorrhage [2]. The study process and schedule for outcome assessment are presented in Fig. 1 and Table 1.

**Table 1:**
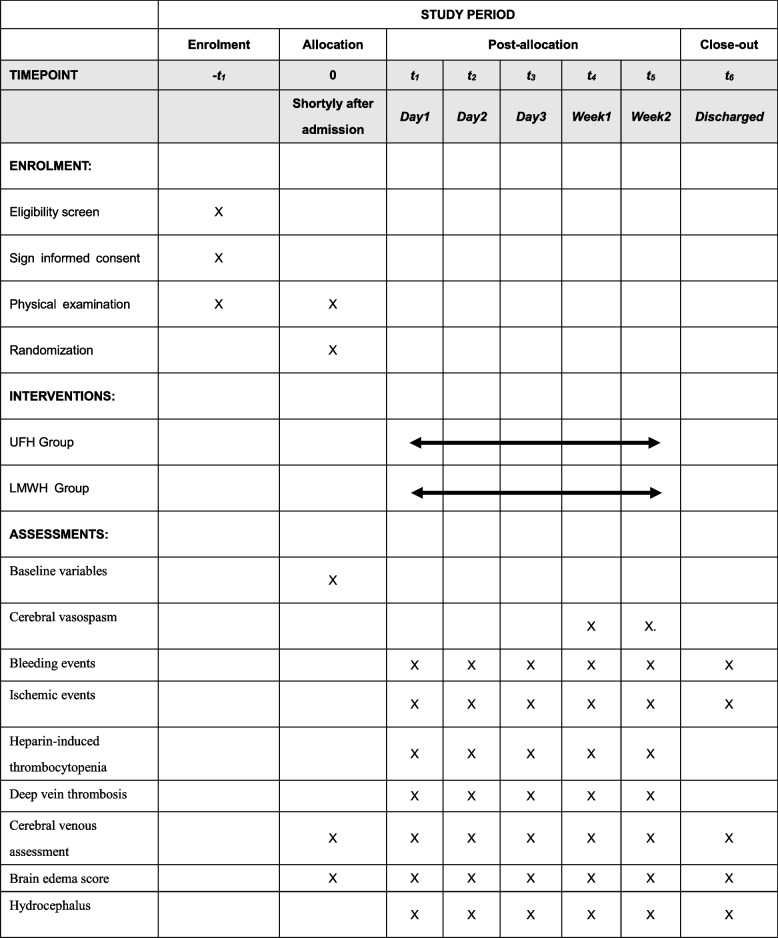
The schedule of enrolment, interventions, and assessments

### Inclusion criteria

- Aged 18–70 years
- Diagnosis of aSAH from anterior communication circulation single aneurysm
- Admission to the hospital within 72 h of the onset of symptoms
- Underwent the endovascular treatment of the ruptured aneurysm
- Written informed consent obtained from the patients

### Exclusion criteria

- Posterior communication circulation aneurysm
- Contraindication to heparin therapy, such as active bleeding or severe thrombocytopenia
- Renal failure, malnutrition, and malignant tumor
- Pregnant patients

#### Recruitment

In order to ensure the recruitment of a sufficient number of anticipated participants, our research team will collaborate with a network of 27 community health centers affiliated with Baoan People’s Hospital Group. This partnership will enable us to enlist a greater pool of potential eligible patients for inclusion in the study, and suitable participants will be selected based on predetermined inclusion and exclusion criteria. The screening logs will be maintained to document reasons for candidate exclusion. The consent process will follow the guidelines outlined in the Helsinki Declaration and the Good Clinical Practice International Coordinating Committee. Participants will receive an informed consent form that explains the study purpose, procedures, risks, and benefits and will be given adequate time to consider participation. For participants who are unable to provide their own consent, a legally authorized representative will do so on their behalf. Participants who meet the inclusion criteria and provide informed consent will be randomized to one of two groups using software-generated randomization sequences. Group allocation information will be kept confidential for the entire study period and only disclosed in special circumstances to prevent bias.

### Study intervention

#### Randomization and blinding

This is a randomized, double-blind, controlled trial in which participants will be randomly assigned to one of two groups: the UFH group and the LMWH group. Participants will be unaware of their treatment assignment. The study aims to recruit 286 patients, with 143 patients in each group. Randomization will be conducted using a randomization sequence generated by R software. The specific method for randomization is as follows: install and load the rlist package, generate participant IDs, and use the shuffle function to randomize the participant IDs. The randomized ID data will then be arranged in order, with IDs 1–143 assigned to the UFH group and IDs 144–286 assigned to the LMWH group. Randomization will be performed by an assistant who is not involved in the study, and detailed information regarding the allocation will be kept in sealed envelopes labeled with a sequence number and will not be disclosed to participants. Data evaluation and analysis will be conducted by independent researchers under the supervision of an independent statistician. Participants, data-collecting researchers, and follow-up teams are precluded from making group allocations.

Severe adverse events include shock caused by thrombocytopenia and cerebral hemorrhage. If a severe adverse event occurs, the intervention should be discontinued if necessary, and the patient should receive appropriate rescue treatment. Blinding will be opened, and the event will be reported to the institutional review board. Compensation will be provided to subjects who are injured as a result of participating in the trial. Medical data will still be collected and analyzed. If a general adverse event occurs, patients do not need to be unblinded.

### Interventions, administration and duration

As part of the trial design, all enrolled patients will receive intervention following embolization of the aneurysm. The UFH group will start intravenous infusion of UFH 24 h after the embolization procedure, upon confirmation of inactive bleeding through head CT scan. The heparin will be administered at a dose of 8–10 U/kg/h, and APTT values will be monitored twice a day for 7 consecutive days. The infusion rate of heparin will be adjusted based on APTT values to maintain it within the range of 35–45 s. In case of APTT values exceeding or falling below the set range, the infusion rate will be decreased or increased by 1 IU and maintained for 12 h. As per FDA standards for the use of low molecular weight heparin, the UFH group will receive subcutaneous injection of prophylactic dose of LMWH (4000 IU) every 12 h for a duration of 1 week starting 24 h after the aneurysm repair.

All experimental UFH is stored in original packaging at 15–30 °C (protected from light and heat). LMWH is stored at 2–8 °C. After opening for more than 24 h, heparin should be discarded. During the administration process, the nursing staff is trained on medication usage and provided with dosing instructions and adverse reaction management plans for both the UFH and LMWH group. To ensure blinding, any communication between the research staff and participants that may reveal group allocation should be avoided. The identity of the medication provider should also not be disclosed.

During hospitalization, both groups of patients will be closely monitored for vital signs and symptoms in the intensive care unit. Platelet-inhibiting agents, nonsteroidal anti-inflammatory drugs, and oral anticoagulants are prohibited during the patient’s treatment period. Physicians will check for interactions with heparin when using other medications and closely monitor bleeding symptoms, adjusting the dosage of heparin or other medications as needed. All patients will receive oral nimodipine, and the blood volume will be maintained in equilibrium. The intracranial pressure of all patients will be monitored with the transducer. Transcranial Doppler (TCD) ultrasound will be performed daily to evaluate for CV, and a weekly follow-up head CT will be performed. In cases where CV or increased intracranial pressure is suspected, urgent head CT angiogram (CTA) or CT scans will be performed for confirmation.

During the use of heparin, the permissible, required, and prohibited intervention measures should comply with the inclusion and exclusion criteria. The protocol should outline all medications and treatments/interventions allowed during the study period, including emergency medications and any necessary interventions. In addition, any prohibited interventions should be included and, if necessary, a list of prohibited medications should be provided in the appendix.

We will make every effort to improve compliance with the intervention protocol, including training for nurses and other relevant researchers. The list of treatments will be used to ensure proper implementation of the intervention measures.

### Intervention discontinuation

Throughout the entire period of the trial, we will establish a data and safety monitoring committee (DSMC) to oversee the progress of the clinical trial, establish an adverse event reporting system to record and report any serious adverse events (SAE) or unexpected outcomes, establish an emergency unblinding procedure to address any serious medical issues that may arise during the trial, develop contingency plans to address any unexpected interruptions or delays that may affect the trial process, and take measures to minimize the impact of any possible interruptions. The researchers do not have the right to decide on the withdrawal of participants, and all discontinuation and deviations from the study protocol will be recorded and reported to the DSMC, regulatory agencies, and the study sponsor.

### Confidentiality

All medical data and personal information of the participants will be kept confidential in accordance with international guidelines and regulations. Only designated members of the research team will have access to participant data, and access will be limited to the information necessary to perform their specific duties. Participant identification codes will be used instead of participant names to ensure anonymity and confidentiality. Data sharing and confidentiality agreements will be developed as needed to protect the confidentiality of research data, analytical methods used, and results obtained. Participants’ medical data and personal information will be encrypted, password protected, or stored offline as needed to ensure data security. All documents related to the study, including informed consent forms and participant medical records, will be securely stored in designated locations to ensure confidentiality.

### Outcome assessments

#### Baseline assessment

Baseline variables encompass demographic information (gender, age, ethnicity, prior medical history, personal life history), comorbidities, vital signs (blood pressure, heart rate, respiratory rate), concomitant medication, surgical intervention (e.g., decompressive craniectomy), and results of the laboratory tests. In addition, Hunt-Hess scales [14] and World Federation of Neurosurgery Societies (WFNS) [15] grade were used to assess the severity of clinical symptoms. Modified Fisher score [16] was employed to evaluate the degree of CV. Assessment of functional independence was performed by the Modified Rankin Scale (mRS) [17]. All clinical baseline data were conducted upon admission and during hospitalization by two researchers who were unaware of the group assignment.

### Primary and secondary outcomes

The primary outcome was the incidence of CV, while secondary outcomes included the incidence of bleeding events, ischemic events, HIT, deep vein thrombosis, cerebral venous circulation time, brain edema score, and incidence of hydrocephalus. The time points for the primary outcomes are the first and second weeks following the patient’s hospitalization and intervention treatment. The time points.for the secondary outcomes include the first 3 days after hospitalization, the first and second weeks, and the follow-up within 1 year after discharge. The CV was defined as the presence of significant neurological deficits in patients, with TCD ultrasound assess a mean flow velocity of the middle cerebral artery (MCA) exceeding 120 cm/s or a Lindegaard ratio (mean flow velocity of MCA/mean flow velocity of extracranial internal carotid artery) greater than 6. Computed tomography angiography scans showed temporary narrowing of cerebral artery. Regardless of the severity or clinical status of the patient, it was evaluated as CV.

Bleeding events are defined as bleeding that occurs in any part of the body, including intracerebral hemorrhage, gastrointestinal bleeding, and subcutaneous bleeding. According to the standardized bleeding score of Bleeding Academic Research Consortium (BARC), it has 5 types [18]. Type 1 refers to bleeding that is considered insignificant and does not prompt the patient to seek medical attention. Type 2 bleeding includes any obvious sign of hemorrhage that requires medical attention, diagnostic testing, hospitalization, or treatment by a healthcare professional. Type 3 bleeding encompasses clinical, laboratory, and/or imaging evidence of bleeding that requires a specific response from a healthcare provider. Type 4 bleeding is related to coronary artery bypass grafting within 48 h, and type 5 bleeding is fatal. Fatal bleeding can be categorized as intracranial, gastrointestinal, retroperitoneal, pulmonary, pericardial, genitourinary, or other.

Ischemic events include cerebral and cardiac ischemia, with cerebral ischemia encompassing transient ischemic attacks and stroke, while cardiac ischemia includes angina pectoris and myocardial infarction. HIT is defined as a decrease in platelet count of 50% or more after the therapy of heparin. Deep venous thrombosis includes lower limb deep venous thrombosis and pulmonary embolism, which is diagnosed by a respiratory physician based on pulmonary vascular examination and blood D-dimer testing. The diagnose of lower limb deep venous thrombosis is detected by ultrasound. Hydrocephalus is defined as an enlargement of the cerebral ventricles as visualized on a brain CT scan.

Cerebral venous circulation time is defined as the time required for blood to enter the venous sinus through the deep and superficial veins of the brain and then be drained into the jugular vein, including microvascular cerebral circulation time, venous cerebral circulation time, and the PRECISE score. Following S. Wang’s research, the starting point T1 is set as the first image in the sagittal view of digital subtraction angiography (DSA), which depicts the contrast dye within the branches of the middle cerebral cortical arteries [19]. The vein image that shows the peak filling of the parietal cortical vein is set as T2, and the only disappearance of the sigmoid sinus was set as T3. The mCCT is expressed as T2-T1, while vCCT is expressed as T3-T2.

The PRECISE score is based on the venous filling level shown on the head CTA. A score of 2 represents complete filling that is consistent with the degree of filling in the contralateral hemisphere, while a score of 1 represents partial filling, and a score of 0 indicates no filling. The filling difference of the affected hemisphere veins is calculated based on the PRECISE score. A higher score indicates the improved cerebral venous outflow, while a lower score signifies the decreased cerebral venous outflow. A score of 2 will be given for “complete” contrast filling of the opposite hemisphere, while a score of 1 will be given for “partial” (moderate contrast filling). The PRECISE score will be calculated using the composite score of the normal hemisphere SMCV + VOT + VOL + BVR minus the composite score of the lesioned hemisphere SMCV + VOT + VOL + BVR [20].

The assessment of brain edema is mainly based on SEBES score which is mainly based on head CT images and intracranial pressure monitoring values during hospitalization [21]. Head CT scans will be conducted at four stages: at the time of admission (0–2 days after aSAH), after endovascular treatment (4–7 days after aSAH), during hospitalization (7–14 days after aSAH), and before discharge (14–21 days after aSAH). Two blinded neurologists specializing in cerebrovascular diseases will independently evaluate the SEBES value of the patients. The scoring criteria will range from 0 to 4 points. Two pre-determined levels in each hemisphere will be defined, and 1 point will be given for visible sulcal effacement due to the interruption of the gray-white matter connection. Intracranial pressure monitoring will include intracranial ICP monitoring, which is recorded by placing an intracranial probe.

### Sample size estimation

The primary outcome of this study was the incidence of CV. J. Marc’s research indicated that the incidence of CV was 58% and 60% in the groups receiving intravenous unfractionated heparin and subcutaneous low molecular weight heparin, respectively [10]. The hypothesis was that the unfractionated heparin protocol was superior to the subcutaneous low molecular weight heparin injection in reducing the incidence of CV. The sample size of the study was calculated using a bilateral test to achieve a target effect of 0.1 and an α error of 0.05. Considering a dropout rate of 10%, each group would require 228 patients, so a total of 456 participants will be enrolled. The sample size was estimated using PASS (version 15.0.3, NCSS, LLC, Kaysville, Utah, USA).

### Statistical analyses

The data analysis will be carried out with statistical software (SPSS, version 26, SPSS Inc). The covariates between groups will be balanced based on Propensity score matching (PSM). The results of outcomes will be analyzed for differences between the UFH group and LMWH group, using the Student *t*-test, chi-square test, and Mann–Whitney *U* test. For continuous variables, the normality test is estimated using the mean (standard deviation). If parameters are not normally distributed, their median (interquartile range) will be reported. For categorical variables, the proportions of the two groups will be presented. Spearman’s correlation analysis is performed on nonnormal continuous variables. Pearson’s correlation is employed for categorical variables. Variables with differences between the two groups will be analyzed by univariate analysis and multifactor logistic regression. Cox multivariate regression analysis will be used for time-to-events data. All statistical tests are two-sided with *p* < 0.05, crude odds ratio (OR), and confidence intervals (CI) will be calculated. In addition, the number of recruitments, participants lost due to follow-up, violations of the protocols, and other relevant descriptive data will be reported. The missing data will be handled using multiple imputation methodology.

### Safety assessments

To ensure the safety of the trial, it is imperative to closely monitor the patient’s condition, including changes in consciousness and vital signs. Laboratory and imaging tests are also necessary to promptly detect any adverse reactions associated with the drug, such as HIT and cerebral hemorrhage leading to cerebral herniation. Any confirmed adverse outcomes will be reported to the DSMC. In the event of a high risk of severe cerebral hemorrhage and cerebral herniation, emergency craniotomy or hematoma evacuation surgery is necessary. A decrease in platelet count is a direct manifestation of HIT. If a sharp decrease in platelet count is observed, heparin use should be immediately stopped. Common rescue drugs for severe HIT include immunoglobulin and plasma exchange.

### Data safety monitoring board

For this study, we shall establish a DSMC. The DSMC, being independent of the researchers, consists of some clinical experts, statistical specialists, and legal and ethical professionals, as well as data management experts. The clinical experts will be responsible for identifying potential recruits and obtaining their consent. The legal and ethical professionals will oversee the trial, convening 1–2 annual meetings. The trial steering committee (TSC), composed of principal investigators, clinical experts, and sponsor representatives, will primarily focus on trial quality control, supervision, monitoring of trial progress, and ensuring safety. They will provide annual oversight of the trial’s progress.

## Discussion

Heparin alleviates CV by binding to inflammatory proteins, reducing free radical release, and inhibiting endothelin-1 [22] transcription to diminish vascular constriction. It also attenuates the downregulation of potassium ion channels caused by oxidized hemoglobin, reducing the severity of CV. Additionally, heparin inhibits smooth muscle cell proliferation and reduces vascular constriction by inhibiting the NF-κB pathway [23] and vasoconstrictor factors [22].

Heparin has been shown in animal models and in vitro experiments to relax blood vessels, enhance blood flow, and prevent CV in aSAH. Studies have demonstrated the safety and efficacy of LMWH in reducing the risk of ischemia and stroke in aSAH patients [9]. Research by Gebriele Wurm et al. found that enoxaparin, compared to placebo, safely and effectively reduced aSAH-related CV [13]. However, there is a need for more randomized controlled trials comparing LMWH with UFH.

Brain edema plays a crucial role in prognosis and early brain injury monitoring in aSAH. Various factors contribute to cerebral edema, including ischemic injury, inflammation, blood degradation products, microvascular damage, and abnormal autoregulation [24]. Cerebral venous system dysfunction and altered cerebral blood flow, including prolonged cerebral circulation time [25], can contribute to elevated intracranial pressure and the development of cerebral edema. Understanding the impact of cerebral circulation time on cerebral edema is important in assessing and managing aSAH patients [14].

When considering the risk–benefit balance of subcutaneous injection of LMWH and intravenous infusion of UFH, it is important to consider their specific characteristics and applications. The bleeding risk and prevention of ischemic effects of both agents are like two sides of a coin, and it is crucial to find a situation where the benefits outweigh the risks. Monitoring coagulation is a common approach to ensure that the bleeding risk is not increased. Compared to LMWH, UFH has a higher molecular weight, shorter half-life, flexible dosing, and faster onset of action, but it requires close monitoring [26]. The choice between the two in aSAH depends on the individualized needs of the patient.

UFH accelerates the inactivation of factors Xa and IIa by binding to thrombomodulin III, preventing the obstruction of blood flow by thrombin, and reducing the risk of thrombus formation [8]. LMWH also acts by enhancing the inhibitory activity of antithrombin III (ATIII) on factor Xa, preventing thrombin formation, and reducing the risk of clot [27]. Thrombophilia can induce platelet activation and aggregation, as well as the release of thromboxane A2 and serotonin, which promote vasoconstriction and contribute to CV. Clots can also mediate the release of inflammatory mediators, causing endothelial dysfunction and an imbalance between vasoconstrictive and vasodilatory factors, which can also result in vasospasm. In summary, heparin indirectly inhibits the occurrence of CV by reducing thrombus formation through ATIII.

The choice between LMWH and UFH depends on balancing the antithrombotic effect and bleeding risk. Some non-randomized clinical trials have demonstrated that heparin can reduce the occurrence of ischemia in aSAH. The beneficial effects of heparin are influenced by the type of heparin and the duration of administration. However, there is no significant difference in the incidence of CV-related to LMWH and UFH, suggesting the possibility that heparin may exert beneficial effects through mechanisms other than preventing CV. The impact of heparin on cerebral edema and cerebral venous circulation in aSAH has not been studied.

This randomized trial aims to compare the safety and efficacy of UFH and LMWH in aSAH and analyze their effects on CV, brain edema, and cerebral venous circulation.

### Strengths and limitation

It is important to acknowledge that this study has some limitations. First, the study is a single center, which may limit the generalizability of the findings to other populations. Second, the study population is exclusively Asian, which may introduce confounding factors related to ethnicity and genetic factors. Finally, the study will not include patients with posterior circulation aneurysms, which may limit the scope of the study’s findings.

Despite these limitations, this will be the first randomized controlled trial to evaluate the effects of different types of heparin and routes of administration on aSAH. It will provide valuable insights and serve as a reference for future clinical treatment and research in aSAH.

### Ethics and dissemination

The sponsor of this study is the Neurological Center of Shenzhen Baoan People’s Hospital. The contact information for the sponsors is as follows: No. 118, Longjing 2nd Road, Baoan District, Shenzhen, Guangdong Province, China. Postal Code: 518,051. They ensure the provision of adequate medical insurance compensation and bear certain legal liability prior to the commencement of the research. The sponsor played no part in study design; collection, management, analysis, and interpretation of data; writing of the report; and the decision to submit the report for publication.

Ethics approval for this study (BYL20220805) was provided by the Ethics Committee of Shenzhen Baoan People’s Hospital in December 2022. Substantial amendments to the protocol will be submitted to this Ethics Committee for approval. This study protocol was registered on 13 January 2023 at ClinicalTrials.gov, and the ID number is NCT05696639. Any proposed amendments to the agreement will be submitted to the Ethics Committee for approval. All participants will be provided with a participant information sheet and a consent form describing the study and sufficient information to make an informed decision about their participation. The consent form must be signed by the participant or a legally acceptable representative and obtained by the research professional designated by the investigator. All treatment regimens received by enrolled patients will be conducted in accordance with the most recent international guidelines and standards. The results of the study will be published in a peer-reviewed journal and presented at international conferences. The principal investigator will retain the ultimate authority over all activities related to the publication and dissemination of the outcomes of the study. Professional writers eschew the usage of inappropriate phrases in their compositions.

## Supplementary Information

**Additional file 1.**

**Additional file 2.**

## Data Availability

At present, no data has been generated. However, the future datasets, study analysis, and statistical code can be obtained from the corresponding author upon a reasonable request, similar to the availability of the current full protocol.
