# Supplementary material for: Effects of low-dose intravenous heparin therapy in aneurysmal subarachnoid hemorrhage: a randomized controlled clinical trial protocol
Source: Trials. 2023 Jul 8;24:447. doi: 10.1186/s13063-023-07493-9 (PMC10329784; doi:10.1186/s13063-023-07493-9)
Supplement: Supplementary file 2 — Additional file 2. [file 13063_2023_7493_MOESM2_ESM.docx]

**Research Informed Consent**

**TITLE OF STUDY**

Effects of low-dose intravenous heparin therapy in aneurysmal subarachnoid hemorrhage: a randomized controlled clinical trial protocol

**PRIMARY RESEARCHER**

Name – Yifan Zhang

Department - Neurology

Address - China City ShenZhen State Guangdong

Phone - +86-13928491802

Email – yifanhahahah@163.com

**PURPOSE OF STUDY**

After clinical examinations, it has been confirmed that you have suffered from subarachnoid hemorrhage caused by cerebral aneurysm. According to your specific condition, you require further vascular intervention treatment. We sincerely hope that you can participate in a study titled “Effects of low-dose intravenous heparin therapy in aneurysmal subarachnoid hemorrhage: a randomized controlled clinical trial protocol”. This study is supported by Youth Scientific Research Fund Project of Shenzhen BaoAn People's Hospital. This research has been approved by the Ethics Committee of our hospital. This study complied with the principles of the Declaration of Helsinki and complied with medical ethics. This study is based on the principle of voluntary participation, and you have the right to withdraw at any stage of the study, which will not affect your future examination and treatment.

The main reason for doing this study is to help answer the following research question:

- Compare effects of low-dose unfractionated heparin and subcutaneous injection of low molecular weight heparin on aneurysmal subarachnoid hemorrhage.
- Assess the effects of low-dose unfractionated heparin on cerebral vasospasm, cerebral edema, and cerebral venous circulation in patients with aSAH.

**PROCEDURES**

After the embolization procedure, you will undergo routine lateral angiography to determine the status of the cortical venous imaging and routine angiography in the working position to determine the degree of aneurysm embolization. Postoperatively, routine treatment such as analgesia, sedation, anti-spasm, dehydration, and external ventricular drainage will be given to release hemorrhagic cerebrospinal fluid and record intracranial pressure (ICP) values. The patient's head CT will be reviewed daily or as necessary depending on the condition to understand the status of brain edema, and the interval between examinations may be extended after the patient's condition stabilizes.

**RISKS**

Collecting imaging data during your interventional treatment will not increase any additional risks to your treatment process. Throughout your entire treatment process, due to the comprehensive imaging and blood tests required, this may occupy some of your time and may cause inconvenience or trouble. Some patients may experience discomfort such as pain during blood collection, but this can be alleviated by the doctor's treatment. If you experience any discomfort during the examination period or if your condition changes, regardless of whether it is related to the study, you should inform your doctor immediately, and he/she will make an appropriate medical judgment and give appropriate medical treatment.

**BENEFITS**

You will receive free comprehensive cerebral vascular evaluation and follow-up telephone consultations from the physician.

**CONFIDENTIALITY**

Please do not write any identifying information.

Every effort will be made by the researcher to preserve your confidentiality including the following:

- Assigning code names/numbers for participants that will be used on all research notes and documents
- Keeping notes, interview transcriptions, and any other identifying participant information in a locked file cabinet in the personal possession of the researcher.

Participant data will be kept confidential except in cases where the researcher is legally obligated to report specific incidents. These incidents include, but may not be limited to, incidents of abuse and suicide risk.

**COMPENSATION**

You will receive free comprehensive cerebral vascular evaluation.

**CONTACT INFORMATION**

If you have questions at any time about this study, or you experience adverse effects as the result of participating in this study, you may contact the researcher whose contact information is provided on the first page. If you have questions regarding your rights as a research participant, or if problems arise which you do not feel you can discuss with the Primary Researcher directly by telephone at +86-13928491802 or at the following email address yifanhahahah@163.com.

**VOLUNTARY PARTICIPATION**

Your participation in this study is voluntary. It is up to you to decide whether or not to take part in this study. If you decide to take part in this study, you will be asked to sign a consent form. After you sign the consent form, you are still free to withdraw at any time and without giving a reason. Withdrawing from this study will not affect the relationship you have, if any, with the researcher. If you withdraw from the study before data collection is completed, your data will be returned to you or destroyed.

**CONSENT**

I have read and I understand the provided information and have had the opportunity to ask questions. I understand that my participation is voluntary and that I am free to withdraw at any time, without giving a reason and without cost. I understand that I will be given a copy of this consent form. I voluntarily agree to take part in this study.

**Participant's Signature** [_____________________________](https://esign.com/) Date __________


**Researcher’s Signature** [_____________________________](https://esign.com/) Date __________
